# Insights from Epidemiology into Dichloromethane and Cancer Risk

**DOI:** 10.3390/ijerph8083380

**Published:** 2011-08-18

**Authors:** Glinda S. Cooper, Cheryl Siegel Scott, Ambuja S. Bale

**Affiliations:** National Center for Environmental Assessment, Office of Research and Development, United States Environmental Protection Agency, Washington, DC 20460, USA; E-Mails: Scott.Cheryl@epa.gov (C.S.S.); Bale.Ambuja@epa.gov (A.S.B.)

**Keywords:** dichloromethane, methylene chloride, solvents, cancer, epidemiology

## Abstract

Dichloromethane (methylene chloride) is a widely used chlorinated solvent. We review the available epidemiology studies (five cohort studies, 13 case-control studies, including seven of hematopoietic cancers), focusing on specific cancer sites. There was little indication of an increased risk of lung cancer in the cohort studies (standardized mortality ratios ranging from 0.46 to 1.21). These cohorts are relatively small, and variable effects (e.g., point estimates ranging from 0.5 to 2.0) were seen for the rarer forms of cancers such as brain cancer and specific hematopoietic cancers. Three large population-based case-control studies of incident non-Hodgkin lymphoma in Europe and the United States observed odds ratios between 1.5 and 2.2 with dichloromethane exposure (ever exposed or highest category of exposure), with higher risk seen in specific subsets of disease. More limited indications of associations with brain cancer, breast cancer, and liver and biliary cancer were also seen in this collection of studies. Existing cohort studies, given their size and uneven exposure information, are unlikely to resolve questions of cancer risks and dichloromethane exposure. More promising approaches are population-based case-control studies of incident disease, and the combination of data from such studies, with robust exposure assessments that include detailed occupational information and exposure assignment based on industry-wide surveys or direct exposure measurements.

## 1. Introduction

Dichloromethane (methylene chloride) has been used extensively as a paint stripper and metal degreaser and as an extraction solvent in the food industry (e.g., in decaffeination of coffee), in the pharmaceutical industry, and in the production of cellulose triacetate film and fiber. Production and use in the United States peaked around 1980, with a production capacity of 830 million pounds per year [1], decreasing more recently to 350 to 650 million pounds per year [2].

Concerns relating to the carcinogenic potential of dichloromethane arose in the 1980s, based in large part on results from a 2-year inhalation exposure experiment conducted by the National Toxicology Program (NTP), in which dichloromethane exposure resulted in an increased incidence of liver and lung tumors in male and female B6C3F_1_ mice [3,4]. Metabolism of dichloromethane is hypothesized to involve two primary pathways: a CYP2E1 dependent oxidative pathway producing carbon monoxide, and a glutathione-*S* transferase-theta 1 (GST-T1)-catalyzed pathway resulting in the production of two highly reactive intermediates, formaldehyde and *S*-(chloromethyl)glutathione, and carbon dioxide [5–7]. The proportion of dichloromethane metabolized *via* the GST pathway increases at higher exposures. Although GST is considered a detoxification pathway for many chemicals, in the case of dichloromethane it is the GST pathway that has been most strongly implicated in genotoxicity and carcinogenicity [8–15].

The database of epidemiology studies focusing specifically on dichloromethane has expanded considerably with the addition of recent studies of hematopoietic cancers conducted in Europe [16–18] and North America [19–22]. The purpose of this paper is to review the currently available epidemiology studies, focusing on specific cancer sites reported in rodent bioassays and identified through a review of the dichloromethane literature, and highlighting issues that could be addressed in future research.

## 2. Methods

We searched the MEDLINE database (last accessed on 30 March 2011) for epidemiologic studies related to dichloromethane and cancer risk, using dichloromethane, methylene chloride, cohort and case-control as search terms. References within relevant reports were also reviewed, and through this process we identified three papers [16,17,20] that were not found through the MEDLINE search strategy because dichloromethane was not used as an indexing term (*i.e.*, the abstract and key words did not include dichloromethane, but dichloromethane-specific data were presented in the analysis). We supplemented this search with a review of cancer epidemiology studies with data on trichloroethylene and tetrachloroethylene, two related solvents with extensive epidemiology databases, to identify any other papers evaluating cancer risk from dichloromethane exposure that had been missed.

Eighteen papers based on epidemiologic studies of cancer risk were identified and included in this evaluation: four cohorts for which the primary solvent exposure was to dichloromethane, one large cohort of civilian employees at a military base with exposures to a variety of solvents, and 13 case-control studies of specific cancers with data on dichloromethane exposure. Publications based on shorter follow-up periods of some of the cohort studies were also identified and reviewed [23–28]. Because our focus was dichloromethane-specific results, we did not include studies that only provided estimates of general categories of solvents (e.g., any solvent, or chlorinated solvents).

## 3. Results

### 3.1. Cohort Studies

No cohort studies examining cancer incidence and dichloromethane exposure were identified in the literature search; cohort mortality studies with cancer data are summarized in Table 1. Two cohorts were conducted among cellulose triacetate film manufacturing workers in New York State and the United Kingdom, with approximately 1,000 to 1,500 male workers and mean exposure concentrations of 20 to 40 ppm [29,30]. Two other cohort studies were conducted among workers in cellulose triacetate fiber plants in South Carolina and Maryland [28,31,32]. The two fiber plant studies involved higher dichloromethane exposure, and the study in Maryland [32] contained twice as many workers but a shorter mean follow-up period, compared with the two studies of film manufacturing workers. In each of these four studies, the relatively small number of deaths greatly limits their ability to provide insights regarding site-specific cancers other than lung.

Overall, there is little indication of an increased risk of lung cancer among these studies, with estimated SMRs generally <1.0 (ranging from 0.46 to 1.21). One study included discussion of smoking history, obtained from a survey at the facility, that indicated that smoking rates were similar in the workers compared with the general population; thus it is unlikely that differences in smoking could be masking an effect of dichloromethane [29]. Only one of the cohort studies reported an increased risk of liver or biliary tract cancer. In the latest follow-up of the South Carolina fiber plant cohort, the SMR for liver and bile duct cancer, based on four observed cases, was 2.98 (95% CI 0.81–7.63), lower than the SMR of 5.75 (95% CI 1.82–13.8) that was reported in the 1990 analysis based on these same four cases but on a shorter follow-up period (and thus lower number of expected cases) [28,31]. Three of these cases were biliary tract cancers. Elevated SMRs (point estimates greater than 1.0) were seen for brain cancer in both film production cohorts: SMR = 2.16 in New York [29] and 1.45 in the United Kingdom [30], with more mixed results seen in the fiber production cohorts. These estimates are based on a small number of observations (ranging from 1 to 6) and so are relatively imprecise. Although estimates of associations for leukemia (ICD-9 codes 204, 208) were available for all of the film and fiber cohort studies, only one study [29] provided data for non-Hodgkin lymphoma (ICD-9 code 200, 202; SMR 0.49, 95% CI 0.06–1.76, based on two observed cases). The results for leukemia were based on between 0 and 8 observed cases, with one SMR suggesting a doubling of risk (SMR 2.04, 95% CI 0.88–4.03) [29], and the other estimates approximately equal to or less than 1.0. None of the studies provided data on leukemia subtype.

Radican *et al*. is the latest follow-up of a large cohort study of 14,000 civilian workers, 1,222 of whom were exposed to dichloromethane, employed at Hill Air Force Base in Utah for at least 1 year from 1952 to 1956, with follow-up through 2000 [26,27,33,34]. The most detailed exposure assessment was done for trichloroethylene, the primary focus of the study. Dichloromethane, one of 25 other exposures analyzed, was classified as a dichotomous exposure (ever exposed, never exposed), and dichloromethane associations were reported for only three cancer sites (non-Hodgkin lymphoma, multiple myeloma and female breast cancer) [33]. The rate ratios for non-Hodgkin lymphoma and multiple myeloma in relation to dichloromethane in men were 2.2 (95% CI 0.76–5.42) and 2.58 (95% CI 0.86–7.72), respectively. These rate ratios (particularly those for multiple myeloma) were higher than those for any of the other chemicals examined; the next highest observed rate ratios for multiple myeloma were 2.1, 2.0, and 2.0 for *o*-dichlorobenzene, Freon, and the “other alcohols” category, respectively. No cases of either of these cancers were observed in women with dichloromethane exposure, but the rate ratio for breast cancer in women was 2.35 (95% CI 0.98–5.65). Associations of similar magnitude or higher (rate ratios of 2.3–2.8) were also seen between breast cancer and some other exposures (Freon, solder flux, isopropyl alcohol, and 1,1,1-trichloroethane). All of these risk estimates were slightly attenuated from the estimates in the earlier examination of this cohort (with follow-up to 1990) described by Blair *et al*. [27].

The cohort studies with the strongest design are the two triacetate film base production cohorts (Cohort 1 in New York and the United Kingdom cohort, reported in [29] and [30], respectively. These are the cohorts with the most extensive exposure assessment information, and that considered exposure level and duration (as summarized by cumulative exposure) in the analysis. The start of eligibility for cohort entrance corresponds with the beginning of the time when the exposure potential at the work site began, and the follow-up period is relatively long (mean >25 years). Hearne and Pifer [29] also included an analysis of a “Cohort 2”, based on 1,013 men employed at least one year between 1964 and 1970. There is some overlap between Cohort 1 and Cohort 2; 707 men were included in both cohorts. Cohort 2 was the focus of previous analyses by Friedlander *et al*. [23] and Hearne *et al*. [24,25]. It is not an inception cohort, and would have missed anyone leaving, possibly because of illness or death, before 1964).

Although the exposure levels in the cohorts involved in cellulose triacetate fiber production were higher than those of the film production cohorts, the duration of exposure was relatively short in the South Carolina cohort (56% < 5 years) [31]. In addition, detailed work history information was only available for 475 (37%) of the workers [28], and it is not clear how the exposure assessment was applied to workers with missing job history data. In the Maryland triacetate fiber production plant, duration of exposure was not reported [32], and was not considered in the analysis. Also, the cohort began in 1970, even though production began in 1955, and the missing personnel records made it impossible to recreate an inception cohort. The exposure assessment in the study of civilian Air Force base workers [33] allowed for only a dichotomized classification of dichloromethane and other solvents (with the exception of trichloroethylene). For the breast cancer results, the magnitude of the risk estimates for some of the other solvents (*i.e.*, associations similar to or stronger than that seen with dichloromethane) makes it difficult to determine from the available data how much of the increased risk seen with dichloromethane could be accounted for by potential confounding. The use of mortality rather than incidence data, which is of particular concern for cancers with a relatively good survival rate, such as non-Hodgkin lymphoma, is another limitation of all of these cohort studies.

### 3.2. Case-Control Studies

Case-control studies offer the potential for increased statistical power for assessing associations with relatively rare cancers. No case-control studies of liver or lung cancer and dichloromethane exposure were identified in the literature search. Six recent case-control studies of dichloromethane exposure and hematopoietic cancers in adults are summarized in Table 2; this Table also includes one study of dichloromethane exposure and childhood leukemia. These are all population-based studies based on incident cases identified through cancer registries, with the number of cases ranging from 180 [22] to 1,428 [17]. No association was seen in the study of adult leukemia (ICD-9 204,208) in Italy [16], although the results for the subtype of chronic lymphatic leukemia (ICD-9 204.1), based on 150 cases, suggest a possible association in the higher intensity exposure category. This subset was also included in the analysis of non-Hodgkin lymphoma cases presented in Miligi *et al*. [17], grouped within the subtype of small-cell lymphocytic lymphoma. The three studies of non-Hodgkin lymphoma in Germany, Italy and Connecticut observed ORs between 1.5 and 2.2 with dichloromethane exposure (ever exposed, or highest category of exposure) [17–20]. In studies that reported more detailed results, there was also some evidence of higher risk among specific subsets of disease, including small-cell lymphocytic lymphoma [17] and diffuse large B-cell lymphoma [19].

Two studies included multiple myeloma cases, but in one of these only four cases were exposed to dichloromethane so effect estimates were not presented [16]. In the study by Gold *et al*., 180 multiple myeloma cases were selected from two Surveillance, Epidemiology and End Results (SEER) cancer registries [22]. One set of analyses included any possible exposure among the exposed, and a second set of analyses included the low confidence exposure jobs with the unexposed group. In general, somewhat stronger associations or patterns were seen in the second analyses. The OR for ever exposed was 1.5 (95% CI 0.9–2.3) in the first analysis and 2.0 (95% CI 1.2–3.2) with the reclassification of the low confidence jobs. In the second set of analyses, a non-monotonic increasing trend was seen with duration of exposure (OR 1.0, 2.0, 1.1, 2.7, and 2.1, respectively, in the unexposed, 1–4, 5–7, 8–24, and 25–47 years duration groups, trend p = 0.01). Similar patterns were seen with cumulative exposure (trend p = 0.08) and cumulative exposure lagged by 10 years (trend p = 0.06).

In the only study of childhood acute lymphoblastic leukemia, a weak association was seen between any maternal dichloromethane exposure during the 2 years before pregnancy up to the birth (OR 1.34 [95% CI 0.54–3.34]); results were similar when limited to exposures during pregnancy [21]. Stronger associations were seen with probable or definite exposure (OR 3.22 [95% CI 0.88–11.7]) compared with possible or no exposure. The estimates for categories based on concentration and frequency were similar but there was no evidence for an increasing risk with increasing exposure level.

Each of these case-control studies obtained, using a structured interview format, detailed information about all jobs held (or, in the case of the study of childhood leukemia, jobs held in the two years before and during the pregnancy), rather than just the usual or most recent job. This information includes job and industry titles in addition to description of tasks and materials, and was used in conjunction with a job exposure matrix developed to assess intensity and probability of exposure, taking into account temporal changes in solvent use within specific types of workplaces. This exposure assessment procedure is based in large part on expert judgment, for example, by industrial hygienists familiar with specific types of workplaces, and is conducted blinded to case-control status. Five of the studies included supplemental job-specific and industry-specific questionnaire modules focusing on potential exposure to specific solvents [16–18,21,22]. The structured and detailed interview format reduces the potential for a differential reporting of exposures by study participants. The addition of specific questionnaire modules designed to obtain more detailed information regarding tasks and exposure conditions also improves the reliability of the assessment.

Case-control studies of dichloromethane exposure and other types of cancers are summarized in Table 3. There was little evidence of associations in the studies of breast cancer [36], pancreatic cancer [37], kidney cancer [38] or rectal cancer [39]. Two case-control studies of dichloromethane exposure and brain cancer have been conducted [40,41]. The stronger of these studies in terms of detail of exposure data was the Heineman *et al*. study based on cases identified using death certificates from southern Louisiana, northern New Jersey, and the Philadelphia area, with confirmation of diagnosis using hospital records [41]. Controls (frequency matched to cases by age, year of death, and study area) were randomly selected from the death certificates of white males who died of causes other than brain tumors, cerebrovascular disease, epilepsy, suicide, and homicide. Data pertaining to lifetime job history were collected from next-of-kin interviews for cases and controls. There was a trend of increasing risk with increasing probability of exposure to dichloromethane (OR = 1.0, 95% CI 0.7–1.6, for low probability; OR = 1.6, 95% CI 0.8–3.0, for medium probability; OR = 2.4, 95% CI 1.0–5.9, for high probability compared with the referent group of unexposed men; trend p-value < 0.05). The highest risk was seen with the combination of long duration (>20 years) and high intensity (or high probability) exposure. Similar results were seen in additional analyses controlling for age, study area, employment in electronics occupations and industries, and exposure to carbon tetrachloride, tetrachloroethylene, and trichloroethylene. In the analyses adjusting for these other exposures, only dichloromethane exhibited a trend with increasing probability of exposure.

As with the case-control studies of hematopoietic cancers, each of these studies used a job exposure matrix based on expert judgment for the classification of various dimensions of exposure (e.g., probability, intensity, frequency). The job exposure matrix approach is based on work in the 1990s by the National Cancer Institute [42,43]. There is a considerable range among these studies, however, in the detail and quality of the exposure information upon which the classification scheme could be applied, from death certificate occupation data [36,37,40], to interview-based information on most recent and usual jobs [38], to a lifetime job history [39,41]. Dell *et al*. noted the limitation of the lack of direct exposure measures in this type of assessment, and the difficulty in categorizing jobs with occasional exposure to a specific solvent [44]. The reliability of this procedure is likely to have been improved in the Heineman *et al*. study [41] by the use of more detailed coding of specific jobs within the industry and occupation code categories to distinguish those of particular relevance to a specific exposure (e.g., production of paint removers within the broader category of production of paints, varnishes, lacquers, enamels, and allied products) [43].

## 4. Discussion

Liver cancer has been a major focus of research on dichloromethane. No data from a case-control study of liver cancer are available pertaining to dichloromethane exposure. The cohort study with the higher exposures, the South Carolina triacetate fiber production plant, suggested an increased risk of liver cancer [28,31]. The SMR for liver and biliary tract cancer was 2.98 (95% CI 0.81–7.63) in the latest update of this cohort. This observation was based on four cases; three of these cases were biliary tract cancers, a very rare form of cancer (expected number estimated as 0.15 cases in [28]). No other cohort study has reported an increased risk of liver cancer mortality, although it should be noted that there is no other inception cohort study of a population with exposure levels similar to those of the South Carolina plant.

In the 2-year NTP inhalation exposure study in B6C3F_1_ mice (exposure concentrations 0, 2,000, and 4,000 ppm), the liver tumor incidence in male mice increased from 44% in controls to 66% at 4,000 ppm; in females, the incidence rose from 6% to 83% across dose groups (both mortality-adjusted trend p-values < 0.001) [3,4]. The results of an oral exposure (drinking water) study in B6C3F_1_ mice are more ambiguous, however [45,46]. There was no indication of an increased incidence of liver tumors in female mice in this study. In males, the incidence of hepatocellular adenomas or carcinomas was 18% and 20% in each of two control groups (combined incidence, 19%), increasing to 26%, 30%, 31%, and 28% in the 60, 125, 185 and 250 mg/kg-day groups, respectively. Serota *et al*. concluded that there was no dose-related trend and that there were no significant pair-wise differences with the controls, but other interpretations are also supported by the results [46]. Although not provided by Serota *et al*. [46], the statistical results are presented in the full report of the study (Hazleton Laboratories, [45]): the trend p-value was 0.058; p-values for the pair-wise comparisons with the combined control group were p = 0.071, 0.023, 0.019, and 0.036 for the 50, 125, 185, and 250 mg/kg per day dose groups, respectively. None of the chronic exposure studies in rats have shown a relation between dichloromethane exposure and liver or lung tumors [3,45,47–49].

The relevance of the bioassay studies of dichloromethane in mice to humans in low-exposure scenarios has been questioned, given the high exposure conditions of the genotoxicity studies and animal bioassays, the high background rates of liver cancer in male B6C3F_1_ mice, and the relatively high GST activity in mice [50]. Comparisons in mice, rats, and humans of GST enzyme activity in liver and lung tissues indicate a much higher activity in mice. In liver tissue samples, mean GST-T1 activity was 29.7, 18.2, 3.70, 1.60 nmol/min per mg protein in female mice, male mice, rats, and human GST-high conjugator groups, respectively [51]. Another potentially relevant interspecies difference is the localization of GST-T1 within cells. In the mouse, localization is seen in the nuclei of hepatocytes and bile-duct epithelium, while the rat liver does not show preferential nuclear localization of GST-T1. In human liver tissue, some hepatocytes show nuclear localization of GST-T1 and others show localization in cytoplasm, as well as in nuclei of bile duct epithelial cells [52,53].

Consideration of metabolic polymorphisms, both in CYP and GST pathways, however, is important and may modulate susceptibility, as has been shown for trichloroethylene [54]. GST-T1 is expressed at a variety of sites in addition to the liver and lung, including mammary tissue [55], brain [56], and peripheral lymphoctyes [8]. The extent of GSH conjugation and presence of polymorphic phenotypes in these tissues may be significant in understanding the sites of action of dichloromethane, and potential differences in site concordance between species.

The cohort studies pertaining to brain cancer risk are statistically underpowered given the few observed cases, 1 to 6 deaths, and their variable findings are not surprising. The Heineman *et al*. study, the stronger of the two brain cancer case-control studies in terms of exposure assessment strategy and confirmation of diagnosis, reported relatively strong trends (p < 0.05) wih increasing probability, duration, and intensity measures of exposure, and with the combination of high intensity (or probability) and long (>20 years) duration of exposure (OR 6.1, 95% CI 1.5–28.3) [41]. These strong trends were not seen with the cumulative exposure metric. The difference in patterns seen with cumulative compared to other exposure metrics may reflect a more valid measure of relevant exposures in the brain from the intensity measure, as suggested by the study in rats reported by Savolainen *et al*. in which dichloromethane levels in the brain were much higher with a higher intensity exposure scenario compared with a constant exposure period with an equivalent time-weighted average [57]. A statistically significant increased incidence of brain or central nervous system tumors has not been observed in any of the animal cancer bioassays, but a 2-year study using relatively low exposure levels (0, 50, 200, and 500 ppm) in Sprague-Dawley rats observed a total of six astrocytoma or glioma (mixed glial cell) tumors in the exposed groups (in females, the incidence was 0, 0, 0, and 2 in the 0, 50, 200, and 500 ppm exposure groups, respectively; in males, the incidence was 0, 1, 2, and 1 in the 0, 50, 200, and 500 ppm exposure groups, respectively; sample size of each group was 70 rats) [49]. These tumors are exceedingly rare in rats, and there are few examples of statistically significant trends in animal bioassays [58].

Large population-based case-control studies of incident non-Hodgkin lymphoma or multiple myeloma in Germany [18], Italy [17] and the United States [19,20,22] observed ORs between 1.5 and 2.2 with dichloromethane exposure (ever exposed, or highest category of exposure), with higher risk among specific subsets of disease. An extensive exposure assessment protocol was used in several of these studies [17,18,22], including job-specific and industry-specific questionnaire modules focusing on potential exposure to specific solvents. Thus although the available epidemiologic studies do not definitively establish an increased cancer risk in relation to dichloromethane exposure, the consistent observations of associations with non-Hodgkin lymphoma indicate that this type of effect is a concern that cannot be dismissed based on available data. Additional studies focusing on specific subtypes of hematopoietic cancers, particularly non-Hodgkin lymphoma, and multiple myeloma, are needed. Childhood leukemia differs from adult-onset hematopoietic cancers with respect to etiologically relevant time window of exposure and potential biological mechanisms [59]. Only one study of childhood leukemia and dichloromethane is available [21]. The results from this study also indicate that further research into this issue is warranted, and would build upon previous research of childhood leukemia and the broader category of parental (paternal or maternal) solvent exposure [60].

Important to any examination of a collection of epidemiology studies are the changes in diagnostic and classification criteria of human lymphoid tumors, particularly non-Hodgkin lymphoma, where classification changes are most significant [61]. A major shift in thinking occurred around 1995 with the Revised European-American Lymphoma (REAL) classification of grouping diseases of the blood and lymphatic tissues along their cell lines compared to previous approaches grouping lymphomas by a cell’s physical characteristics. It was increasingly recognized that some non-Hodgkin lymphomas and corresponding lymphoid leukemias were different phases (solid and circulating) of the same disease entity [62]. Diagnostic and classification criteria may not be uniform across studies. Classification differences hinder comparison of consistency across epidemiologic studies of lymphoid cancers and dichloromethane. In addition, the misclassification of disease subtype would be expected to result in attenuated effect estimates, as it is unlikely to be systematically related to exposure. The cohort studies conducted in the 1990s used ICD-9 or ICD-8 classifications, which do not incorporate some of the concepts of contemporary knowledge of lymphomas that have been used in more recent case-control studies.

Based on this review, a number of suggestions for future epidemiology research can be made. Existing cohort studies, given their size and uneven exposure information, are unlikely to resolve questions of cancer risks and dichloromethane exposure; however, further follow-up in the New York film cohort [29] may provide limited additional information. Given the low incidence of brain, liver and hematological cancers, and the small number of women in these cohorts, more promising are case-control studies of incident cases identified from population-based cancer registries, such as the National Cancer Institute’s SEER registry. Case-control studies should include robust exposure assessments such as those used in the study of Gold *et al*. [22] with detailed occupational information and exposure assignment referencing industry-wide surveys (see Bakke *et al.* [63] and Gold *et al.* [64] for descriptions of assessments of trichloroethylene and tetrachloroethylene, respectively), or methods that incorporate exposure measures in selected scenarios with questionnaire-based information [65]. Review of task-level information and supplemental questionnaire modules, in addition to industry and occupation codes, as was done in the more recent studies of hematological cancers, can provide valuable information that can improve the sensitivity and specificity of an exposure assessment used in case-control studies [66].

It may also be possible to combine data from the case-control studies of hematological cancers we identified, given the similarities in the exposure assessment methodologies used in the studies, to provide more robust estimates of effects in specific subtypes of these cancers, effects based on different exposure metrics, and effects adjusting for other exposures. The large sample size produced by such an aggregation of studies would be needed to examine gene-environment interactions. For dichloromethane, it is the GST-T1 metabolic pathway that is thought to result in genotoxicity and carcinogenicity. Thus higher risk would be expected among individuals with the GST-T1^+/+^ genotype compared with the GST-T1^−/−^ (null) genotype. Effect modification (*i.e.*, higher risk) would also be expected with genetic variants, or with co-exposures with other CYP substrates (e.g., alcohol, some other solvents), that result in lower CYP2E1 activity and thus higher GST-T1 metabolism of dichloromethane. Barry *et al*. reported an interaction between the TT genotype of CYP2E1 rs20760673, dichloromethane exposure, and risk of non-Hodgkin lymphoma (OR 4.42, 95% CI 2.03–9.62) [19], but the functional significance of the variant is not known.

The available data from the large cohort of civilian workers at an Air Force base indicate that a number of solvents, including dichloromethane, may be associated with breast cancer risk [33]. Future studies examining this issue should enable control of reproductive risk factors, through the choice of referent group or adjustment in the analysis. Potential confounding by co-exposures (e.g., other solvents) should also be addressed in breast cancer studies.

In summary, developments in exposure assessment for use in population settings, disease classification, and the creation of large-scale cohorts of people at higher risk for some diseases, such as the Sister Study for breast cancer [67], provide a strong foundation for future epidemiological studies of dichloromethane and other solvents. The insights generated from these studies can contribute greatly to our understanding of disease risk, and to the interpretation of animal and mechanistic studies.

## Figures and Tables

**Table 1.** Summary of cohort studies of cancer risk and dichloromethane exposure.

**First author [reference], location**	**Total n, exposure level (8 hour TWA) ^a^, follow-up period**	**Inclusion criteria, referent group(s)**	**Exposure assessment; outcome assessment**	**Results**
Hearne [29] Cellulose triacetate film base production; New York (“Cohort 1”)	n = 1,311 men; Mean 39 ppm; mean duration, 17 years; follow-up through 1994; mean follow-up, 35 years	Began working after 1945; worked at least 1 year; referent = New York State excluding New York City	Work history (job records) and personal/air monitoring; cumulative exposure based on summation across jobs of duration and average exposure. Death certificate (underlying causes)	SMR (95% CI) (n observed cases): lung cancer 0.75 (0.49–1.09) (27) liver cancer 0.42 (0.01–2.36) (1) brain cancer 2.16 (0.79–4.69) (6) leukemia 2.04 (0.88–4.03) (8) pancreatic 0.92 (0.30–2.14) (5) non-Hodgkin 0.49 (0.06–1.76) (2) multiple myeloma 0.68 (0.01–3.79) (1)
Tomenson [30] Cellulose triacetate film base production; United Kingdom	n = 1,473 men; mean 19 ppm; mean duration, 9 years; follow-up through 1994; mean follow-up, 27 years	Employed anytime between 1946 and 1988; referent = England and Wales	Work history (job records) and personal/air monitoring (30% missing details of work history); cumulative exposure based on summation across jobs of duration and average exposure. Death certificate (underlying causes)	SMR (95% CI) (n observed cases): lung cancer 0.46 (0.29–0.75) (19) liver cancer 0 observed, 1.5 expected brain cancer 1.45 (0.40–3.72) (4) pancreatic 0.68 (0.14–1.99) (3) leukemia 1.11 (0.23–1.87) (3)
Lanes [28,31] Cellulose triacetate fiber production; South Carolina	n = 551 men, 720 women (total n = 1,271); median 140, 280, and 475 ppm in low, moderate, and high groups; 56% <5 years work duration; follow-up through 1990; mean follow-up, ∼28 years	Worked at least 3 mo in the preparation or extrusion areas from 1954 to 1977; referent = York County, South Carolina	Job history data and personal/air monitoring of specific areas (but job history data available for 37%); no analysis by variation in exposure. Death certificate (underlying and contributing causes)	SMR (95% CI) (n observed cases): lung cancer 0.80 (0.43–1.37) (13) liver cancer 2.98 (0.81–7.63) (4) brain cancer **^a^** 0.67 (0.2–3.71) (1) pancreatic 0.83 (0.10–7.63) (2) leukemia **^a^** 0.54 (0.11–1.57) (1)
Gibbs [32] Cellulose triacetate fiber production; Maryland	n = 1,931 men and 978 women (total n = 2,909); 50–100 ppm in low and 350–700 ppm in high exposure; duration not reported; follow-up through 1989; mean follow-up 17 years	Employed on or after January 1, 1970, for at least 3 month (potential exposure began 1955); referent = Allegany County, Maryland	Work history (job records) and personal/air monitoring; divided into “high” (350 to 700 ppm) and “low” (50 to 100 ppm) exposure. **^c^** Death certificate (fields used not stated)	SMR^b^ (n observed cases) in men; women: lung cancer 0.66 (35); 1.21 (11) liver cancer 0.78 (2); 0.0 (0) brain cancer 0.52 (2); 2.74 (2) pancreatic 0.58 (3); 0.52 (1) leukemia**^a^** 1.15 (5); 0.0 (0) breast not reported; 0.92 (10)
Radican [33,34], Air Force Base, Utah (follow-up of [27] and [26])	n = 10,461 men and 3,605 women (total n = 14,066); exposure dichotomized (yes, no); exposure duration not reported; follow-up through 2000; mean follow-up ∼29 years	Employed at least 1 yr from 1952 to 1956 (potential exposure began 1939); internal referent (unexposed workers)	Work history (job records) and industrial hygiene assessment based on work site review (dichotomized exposure); death certificate (underlying and contributing causes)	RR (95% CI), in men: non-Hodgkin’s lymphoma 2.02 (0.76–5.42 (8) multiple myeloma 2.58 (0.86–7.76) (7) RR (95% CI), in women: breast cancer 2.35 (0.98–5.65) (6)

a Data included in a report by Gibbs [35] that included more extensive data for both triacetate fiber cohorts.

b Calculated by summing observed and expected across exposure groups.

c Exposure classification based on a specific job, rather than a summed exposure across all jobs.

The 8-hour threshold limit value before 1975 was 500 ppm, and the current OSHA Permissible Exposure Level (PEL) is 25 ppm.

**Table 2.** Summary of case-control studies of hematopoietic cancer risk and dichloromethane exposure.

**Cancer type, first author [reference], study details**	**Exposure assessment**	**Results^a^**
Leukemia		
Costantini [16], Italy (7 areas) 586 incident cases, 1,278 population-based controls (area population files); 1991–1993; cancer classification based on NCI protocol; ages 20–74 years, men and women, participation rate 85% (cases), 72% (controls)	Job exposure matrix applied to work history (all jobs held at least 5 years) ascertained through interviews, job-specific and industry-specific questionnaires (for solvent-and other chemical-related jobs). Probability and intensity ratings; 10 specific solvents	intensity measure—all leukemia: very low/low OR 0.7 (0.3–1.7) medium/high OR 0.5 (0.1–2.3) Chronic Lymphatic Leukemia: very low/low OR 0.4 (0.1–2.0) medium/high OR 1.6 (0.3–8.6)
Non-Hodgkin lymphoma		
Miligi [17], Italy (8 areas) 1,428 incident cases, 1,530 population-based controls (area population files); 1991–1993; cancer classification based on NCI protocol; ages 20–74 years, men and women, participation rate 83% (cases), 73% (controls)	Job exposure matrix applied to work history (all jobs held at least 5 years) ascertained through interviews, job-specific and industry-specific questionnaires (for solvent-and other chemical-related jobs). Probability and intensity ratings; 10 specific solvents	Intensity measure: very low/low OR 0.9 (0.5–1.6) medium/high OR 1.7 (0.7–4.3) Small Lymphocytic subtype: any exposure OR 3.2 (1.0–10.1)
Seidler [18], Germany (6 areas); 710 incident cases, 710 population-based controls (area population files), 1999–2003; ages 18–80 years, men and women, participation rate 87% (cases), 44% (controls)	Job exposure matrix applied to work history (all jobs held at least 1 year) ascertained through interviews, job-specific and industry-specific questionnaires (for solvent-and other chemical-related jobs). Probability and intensity ratings; 8 specific solvents	Cumulative exposure (ppm-years): 0 OR 1.0 (referent) >0 to ≤26.3 OR 0.4 (0.7–5.2) >26.3 to ≤175 OR 0.8 (0.3–1.9) >175 OR 2.2 (04–11.6)
Wang [20]; Barry [19], Connecticut, 601 incident cases, 717 population-based controls (random digit dialing and Medicare files), 1996–2000; ages 21–84 years, women, participation rate 72% (cases), 69% (random digit dialing controls), 47% (Medicare controls). Barry [19] is limited to 518 cases and 597 controls with blood or buccal cell sample for genotyping	Job exposure matrix applied to work history (all jobs held at least 1 year) ascertained through interviews (job and industry titles, duties). Probability and intensity ratings; 8 specific solvents	Ever exposure: OR 1.5 (1.0–2.3) Little difference in risk by probability or intensity score. Diffuse Large B-cell Lymphoma: OR 2.10 (1.15–3.85) TT genotype of CYP2E1 rs20760673: OR 4.42 (2.03–9.62)
Multiple Myeloma		
Gold [22], Seattle, Washington and Detroit, Michigan (2 SEER sites). 180 incident cases; 481 population-based controls (random digit dialing and Medicare files), 2000–2002; ages 35–74 years, men and women, participation rate 50% (cases), 52% (controls)	Job exposure matrix applied to work history (all jobs held since age 15) ascertained through interviews, job-specific questionnaires (for solvent-related jobs held at least 2 years). Probability, frequency, intensity and confidence ratings; 6 specific solvents	Low confidence jobs as unexposed: Ever exposed OR 2.0 (1.2–3.2) Trends with duration (p = 0.01), cumulative exposure (p = 0.08) and 10-year lagged cumulative exposure (p = 0.06)
Childhood leukemia (acute lymphoblastic leukemia)		
Infante-Rivard [21], Quebec, Canada. 790 incident cases (hospitals—all provinces), 790 population-based controls (government population registries), 1980–2000; cancer based on oncologist or hematologist diagnosis, ages 0–14 years ^a^, boys and girls, participation rate 93% (cases), 86% (controls)	Systematic review of detailed information on all jobs held by the mother from 2 years before pregnancy through birth of the child; 21 individual substances and six mixtures evaluated (mostly solvents); confidence, frequency, and concentration of exposure ratings	Little evidence of association with any exposure, OR 1.34 (0.54, 3.34), but stronger associations with probable or definite, OR 3.22 (0.88, 11.7) (referent group = possible/no exposure) and with combinations of frequency and concentration

a From 1980 to 1993, study was limited to diagnoses of ages 0–9, but this was expanded between 1994 and 2000 to ages 0–14.

**Table 3.** Summary of case-control studies of cancer risk and dichloromethane exposure.

**Cancer type, [reference]**	**Study Details**	**Exposure assessment**	**Results ^a^**
Brain Heineman [41]	Louisiana, New Jersey, Philadelphia; 300 cases, 320 controls (death certificates); 1978–1981; cancer confirmed by hospital records; white men, participation rate 88% (cases), 83% (controls)	Job exposure matrix applied to detailed information on all jobs held (at least 1 year) since age 15, as obtained from next-of-kin interviews; probability, duration, intensity, and cumulative exposure scores; six solvents evaluated	OR 1.3 (0.9–1.8) for any exposure; increased risk with increased probability (trend *p*-value < 0.05, OR 2.4 [1.0–5.9] for high probability), increased duration, increased intensity; strongest effects seen in high probability plus high duration, OR 6.1 (1.1–43.8) or high intensity and high duration, OR 6.1 (1.5–28.3) combinations; no association with cumulative exposure score
Brain Cocco [40]	24 states (United States); 12,980 cases, 51,920 controls (death certificates); 1984–1992; women	Job exposure matrix applied to death certificate occupation; probability, and intensity scores; 11 exposures evaluated	Weak association overall, OR 1.2 (1.1–1.3), no trend with probability or intensity scores
Breast Cantor [36]	24 states (United States); 33,509 cases, 117,794 controls (death certificates); 1984–1989; black and white women	Job exposure matrix applied to death certificate job data, probability, and exposure level; 31 substances evaluated	Little evidence of association with exposure probability; weak association with highest exposure level in whites, OR 1.17 (1.1–1.3) and in blacks, OR 1.46 (1.2–1.7)
Pancreatic Kernan [37]	24 states (United States); 63,037 cases, 252,386 controls (death certificates); 1984–1993; black and white men and women	Job exposure matrix applied to death certificate occupation, probability, and intensity scores; 11 chlorinated solvents and formaldehyde evaluated	Little evidence of associations with intensity or probability
Kidney Dosemeci [38]	Minnesota; 438 incident cases (Minnesota cancer registry), 687 controls (random digit dialing and Medicare records); 1988–1990; cancer confirmed by histology; men and women, participation rate 87% (cases), 86% (controls)	Job exposure matrices applied to most recent and usual job, as ascertained from interviews; nine solvents evaluated	No evidence of increased risk associated with dichloromethane in men, OR 0.85 (0.6–1.2) or women, OR 0.95 (0.4–2.2)
Rectal Dumas [39]	Montreal, Canada; 257 incident cases, 1,295 other cancer controls from 19 hospitals; 533 population-based controls (electoral rolls and random digit dialing), 1979–1985 cancer confirmed by histology; men, participation rate 85% (cases), not reported (other cancer controls), 72% (population-based controls)	Job exposure matrix applied to detailed information on all jobs held, as ascertained from interviews; 294 substances evaluated	Little evidence of an association with any exposure, OR 1.2 (0.5–2.8), but increased risk in a small, “substantial exposure” group, OR 3.8 (1.1–12.2) (using cancer controls; analysis of population controls not given for this exposure)
